# Circulating Gremlin-1 Reflects Age-Associated Metabolic Changes in Women

**DOI:** 10.3390/metabo16020141

**Published:** 2026-02-19

**Authors:** Rahma M. Alyami, Khalid Al-Regaiey

**Affiliations:** 1 Department of Physiology, College of Medicine, King Khalid University, Abha 62421, Saudi Arabia; rmalyami@kku.edu.sa; 2 Department of Physiology, College of Medicine, King Saud University, Riyadh 11461, Saudi Arabia

**Keywords:** Gremlin-1, IGF-1, menopause, obesity, metabolism, estradiol (E2), HbA1c

## Abstract

Background: Menopause is accompanied by hormonal alterations that are closely linked to changes in body composition, insulin sensitivity, and cardiovascular risk in women. Gremlin-1 has recently been identified as an adipokine involved in metabolic and reproductive aging; however, its associations with endocrine and lipid biomarkers across the menopausal transition remain incompletely defined. Objectives: To evaluate the relationships between plasma Gremlin-1 and IGF-1, HDL cholesterol, estradiol, and age in reproductive-aged and postmenopausal women. Methods: This cross-sectional study included 88 women aged 18–65 years, stratified by menopausal status (reproductive-aged vs. postmenopausal). Plasma concentrations of Gremlin-1, growth hormone, IGF-1, insulin, estradiol (E2), glucose, HbA1c, and a standard lipid profile were measured. Results: Plasma Gremlin-1 concentrations were significantly higher in postmenopausal women compared with reproductive-aged women (*p* < 0.001). Age (*p* = 0.013), but not menopause status (*p* = 0.874), was associated with Gremlin-1 levels. Gremlin-1 showed a strong inverse association with IGF-1 (*p* = 0.003) and a negative correlation with HDL cholesterol (*p* = 0.03) in non-obese women; however this association disappeared after adjustment for age. Conclusion: Circulating Gremlin-1 primarily reflects chronological aging and associated endocrine–metabolic changes rather than menopausal status or adiposity per se. While unadjusted associations with metabolic biomarkers are detectable, these relationships are largely attributable to aging. Gremlin-1 may therefore serve as a marker of systemic aging-related endocrine–metabolic remodeling rather than a specific indicator of ovarian aging or adipose tissue dysfunction.

## 1. Introduction

Menopause represents a pivotal physiological transition in a woman’s life, characterized by a marked decline in ovarian function and a reduction in circulating estrogens [1]. These hormonal alterations are strongly implicated in regulating energy metabolism [2], body fat distribution, and insulin sensitivity, and collectively increase the risk of obesity, type 2 diabetes, and cardiovascular disease in postmenopausal women [3]. The menopausal transition is therefore associated with a rise in metabolic dysfunction, posing a challenge for long-term health maintenance in aging women [4].

Gremlin-1 is a bone morphogenetic protein (BMP) antagonist that has been increasingly recognized for its role in adipose tissue biology [5]. Experimental studies indicate that Gremlin-1 influences adipogenesis and may promote pathological tissue fibrosis [6], suggesting a potential link between obesity and metabolic dysregulation, particularly in aging populations [7].

Increased Gremlin-1 levels have been associated with adipose tissue dysfunction [5], insulin resistance [7], and systemic inflammation, processes that are central to the metabolic alterations observed during menopause [8].

Previous studies suggest that Gremlin-1 may play a role in ovarian aging and altered hormonal signaling. It has been shown to regulates the transition from primordial to primary follicles, highlighting its involvement in early follicular development and reproductive senescence [9].

Similarly, elevated Gremlin-1 levels have been reported in women with polycystic ovary syndrome, a condition associated with impaired folliculogenesis and disrupted estrogen activity [10]. These findings are further supported by evidence showing that Gremlin-1 influences granulosa cell proliferation and steroid hormone production in ovarian follicles, suggesting a role in the regulation of ovarian endocrine function [11].

Collectively, these observations suggest that Gremlin-1 may serve as a molecular link between aging-related tissue remodeling, reproductive decline, and metabolic dysregulation in women.

Established metabolic biomarkers, including insulin-like growth factor 1 (IGF-1), estradiol, fasting blood glucose, glycated hemoglobin (HbA1c), and lipid profiles, are widely used to assess cardiometabolic risk [12]. During menopause, declining levels of IGF-1 [13] and estradiol [14] adversely affect insulin sensitivity and lipid metabolism, contributing to an increased risk of metabolic syndrome and cardiovascular disease [14].

Several studies have demonstrated that reduced IGF-1 levels are associated with impaired glucose homeostasis and increased insulin resistance, both of which contribute to the development of type 2 diabetes mellitus. This association is particularly evident in aging populations, where IGF-1 deficiency may exacerbate the metabolic shifts observed during and after menopause [15].

Beyond glucose regulation, IGF-1 plays a critical role in adipose tissue function by modulating lipid storage and adipokine secretion. Deficient IGF-1 signaling has been linked to increased visceral fat accumulation, elevated triglycerides, and unfavorable lipid profiles, which are well-known risk factors for cardiovascular disease [16].

Despite substantial evidence linking menopause to metabolic alterations, the role of novel biomarkers such as Gremlin-1 in this context remains inadequately understood.

This study aims to investigate the association of circulating Gremlin-1 with obesity and a panel of metabolic and hormonal biomarkers in reproductive-aged and postmenopausal women. By delineating these relationships, it seeks to clarify hormonal–metabolic interactions during the menopausal transition and to explore whether Gremlin-1 may represent a useful target for improving metabolic health outcomes in this high-risk population.

## 2. Materials and Methods

### 2.1. Study Design and Subjects

This cross-sectional study was conducted at the Department of Physiology, King Saud University, and at King Saud University Medical City (KSUMC) in Riyadh, Saudi Arabia. Ethical approval was obtained from the Institutional Review Board of the College of Medicine at King Saud University (Approval No. E-22-6933). All participants signed a written informed consent form before taking part in the study. The research followed the ethical guidelines of the Declaration of Helsinki.

Women who attended the KSUMC outpatient clinics and completed all study procedures were eligible for inclusion.

A total of 88 women aged 18–65 years were recruited and divided into reproductive-aged and postmenopausal groups based on menopausal status. Medical history and menopausal information were confirmed using patient records and structured interviews via questionnaires. Menstrual patterns were assessed through semi-structured interviews. Participants were considered reproductive-aged if they reported regular menstrual cycles, and postmenopausal if they had experienced no menstruation for over 12 consecutive months.

Exclusion criteria included pregnancy, acute medical illness, or any ongoing treatment likely to influence study parameters. Women on hormone therapy and those in the perimenopausal transition were not included.

Baseline demographic, anthropometric, and biochemical characteristics of the study population are summarized in Table 1.

### 2.2. Data Collection

Demographic information was collected using a structured and validated questionnaire with items including age, sex, level of education, and medical history. Participants completed the Menopause Rating Scale (MRS) to evaluate menopausal symptoms and quality of life [17].

### 2.3. Biochemical Analysis

Blood samples (5 mL) were collected in the morning after an overnight fast, using tubes with anticoagulant (sodium citrate). Plasma was separated by centrifugation at 1500× *g* for 10 min and stored at –80 °C in aliquots until further analysis.

Plasma concentrations of Gremlin-1 were quantified using ELISA kit (Abbexa, Cambridge, UK; Cat. No: abx151737), according to the manufacturer’s instructions. Briefly, plasma samples and standards were added to 96-well plates pre-coated with specific capture antibodies and incubated at 37 °C for 90 min. After the addition of 100 μL of Detection Reagent A, the plates were incubated for another hour at the same temperature. Following three washes with the supplied buffer, Detection Reagent B was added, and incubation continued for 30 min. Wells were washed five additional times, then treated with TMB substrate for 20 min at 37 °C to allow color development. The reaction was stopped with 50 μL of stop solution, and absorbance was measured at 450 nm using a microplate reader (EL 800, BioTek Instruments, Winooski, VT, USA).

Plasma concentrations of insulin-like growth factor 1 (IGF-1), growth hormone (GH), and estradiol (E2) were measured using electrochemiluminescence immunoassay (ECLIA) technology in the hospital diagnostic laboratories. The principle of ECLIA involves the use of specific antibodies that bind to the target analyte; upon formation of the antigen–antibody complex, an electrochemical reaction triggers light emission, which is then detected by a photomultiplier. The intensity of the emitted light is directly proportional to the analyte concentration. Assays of IGF-1, hGH, and Estradiol (REF 07475918190, 07027486190, and 07027249190, respectively) were analyzed the Cobas 8000 e801 analyzer (Roche Diagnostics, Mannheim, Germany).

Other parameters, such as insulin, glucose, HbA1c, and lipid profile, were retrieved from hospital records corresponding to the same blood sample collection time.

### 2.4. Statistical Analysis

Continuous variables were assessed for normality using visual inspection of histograms and the Shapiro–Wilk test. Normally distributed data are presented as mean ± standard deviation (SD), while non-normally distributed variables are presented as median (interquartile range). Categorical variables are summarized as frequencies and percentages.

Between-group comparisons according to menopausal status were performed using independent-samples *t*-tests for normally distributed continuous variables and the Mann–Whitney U test for non-normally distributed variables. Associations between categorical variables were assessed using the χ^2^ test, as appropriate.

To identify independent determinants of circulating Gremlin-1 levels, multivariable generalized linear models (GLMs) were constructed with Gremlin-1 as the dependent variable. Predictor variables included chronological age, menopausal status, and body mass index (BMI). Additional metabolic variables were explored in preliminary models but were not retained due to lack of statistical significance. Model estimates are reported as beta coefficients with corresponding 95% confidence intervals (CIs).

Pearson correlation analyses were initially used to examine associations between circulating Gremlin-1 and key metabolic biomarkers, including IGF-1 and HDL cholesterol. To evaluate whether these associations differed by adiposity, correlation analyses were subsequently stratified by BMI category (<30 vs. ≥30 kg/m^2^).

Given the strong associations between age, Gremlin-1, and metabolic biomarkers, partial correlation analyses adjusting for chronological age were then performed within each BMI stratum to assess whether observed associations were independent of aging. All correlation analyses were conducted using complete cases for the variables of interest.

All statistical tests were two-tailed, and a *p*-value < 0.05 was considered statistically significant. Statistical analyses were performed using IBM SPSS Statistics (version 29).

## 3. Results

### 3.1. Hormonal and Clinical Biochemical Parameters

According to independent samples *t*-tests, Postmenopausal women exhibited significantly higher plasma Gremlin-1 levels compared to reproductive-aged women

(t(86) = 3.56, *p* = 0.001). Conversely, GH levels were significantly lower in postmenopausal women compared to reproductive-aged women (t(84) = 1.99, *p* = 0.050) (Table 2).

Significant differences in plasma IGF-1 levels were observed between groups, with postmenopausal women exhibiting lower mean IGF-1 levels compared to reproductive-aged women (t(84) = 6.20, *p* < 0.001) (Table 2).

The women in reproductive-age had measured significantly higher mean estradiol levels on average compared to those in their post-menopause (t(86) = 5.47, *p* < 0.001).

Metabolic markers showed notable differences, postmenopausal women had significantly higher fasting blood glucose (FBG) (t(86) = 2.46, *p* = 0.016) and HbA1c (t(86) = 3.68, *p* < 0.001).

Lipid panel differences were modest. Triglyceride (TG) levels were significantly higher in postmenopausal women (t(86) = 2.17, *p* = 0.036), whereas no statistically significant differences were observed for other lipid parameters, including HDL and LDL (Table 2).

### 3.2. Analysis of Mean Gremlin-1 Level Score

A Generalized Linear Model was fitted to investigate predictors of plasma Gremlin-1 levels among women, using a normal distribution and identity link function. The model included age as independent variable. The analysis revealed that age was significantly associated with Gremlin-1 levels (β = 5.97, 95% CI [1.30, 10.63], *p* = 0.013), suggesting that Gremlin-1 concentrations increase with age. However, menopausal status was not a significant predictor (β = 9.86, *p* = 0.874), indicating no independent effect of menopausal transition on Gremlin-1 after adjusting for age. This suggests that the age-related rise in Gremlin-1 is not meaningfully moderated by menopausal state. The study other measured predictor independent variables were also tested in iterative competing models and were found to be insignificantly correlated with the women’s mean Gremlin-1 plasma level (Table 3).

### 3.3. BMI-Stratified Correlation Analysis

To examine whether associations between circulating Gremlin-1 and metabolic biomarkers differed by adiposity, correlation analyses were stratified by BMI category (<30 vs. ≥30 kg/m^2^). In women with BMI < 30 kg/m^2^, Gremlin-1 showed a strong inverse association with IGF-1 (r = −0.447, *p* = 0.003) and a moderate inverse association with HDL cholesterol (r = −0.340, *p* = 0.028). In contrast, these associations were attenuated and not statistically significant among women with BMI ≥ 30 kg/m^2^ (Table 4).

### 3.4. Age-Adjusted Stratified Correlation Analysis

To evaluate whether the associations between circulating Gremlin-1 and metabolic biomarkers were independent of chronological aging and modified by adiposity, age-adjusted partial correlation analyses were performed within BMI strata (<30 and ≥30 kg/m^2^). After adjustment for age, Gremlin-1 was not significantly associated with IGF-1 or HDL cholesterol in either BMI category (Table 5). These findings indicate that the unadjusted correlations observed in earlier analyses were largely attributable to shared age-related variance rather than independent effects of Gremlin-1.

## 4. Discussion

This study investigated the relationship between circulating Gremlin-1, obesity, and metabolic biomarkers in reproductive-aged and postmenopausal women [18]. Menopause represents a major physiological transition accompanied by profound hormonal changes that influence body composition, insulin sensitivity, and cardiometabolic risk [14,19]. By assessing plasma Gremlin-1 alongside IGF-1, estradiol, glycemic indices, and lipid parameters, we sought to better characterize how reproductive aging and adiposity intersect to shape metabolic health in women.

We observed markedly higher plasma Gremlin-1 concentrations in postmenopausal compared with reproductive-aged women. However, multivariable generalized linear analysis revealed that age, rather than menopause, was associated with Gremlin-1 elevation. This finding likely reflects the combined impact of reproductive aging, altered ovarian signaling, and menopause-associated metabolic changes [20]. Gremlin-1 functions as a bone morphogenetic protein antagonist and plays a recognized role in ovarian follicular development. Experimental studies have shown that members of the Gremlin family regulate early follicle activation and interact with BMP-related pathways involved in ovarian reserve. In this context, the elevation of circulating Gremlin-1 after menopause may partly mirror age-related changes in ovarian biology [9]. Moreover, Metabolic factors are also likely to contribute. In previous work, we reported higher Gremlin-1 levels in women with type 2 diabetes, where Gremlin-1 correlated positively with adiposity, HbA1c, and indices of insulin resistance [21]. Given that menopause is commonly accompanied by increased fat mass and declining insulin sensitivity [22,23], Gremlin-1 levels increase progressively with advancing age despite physiological changes typically associated with declining estrogen levels. Taken together, postmenopausal status did not significantly predict Gremlin-1 levels, suggesting that estrogen decline alone does not fully explain age-associated upregulation.

In our cohort, circulating GH and IGF-1 levels were lower in postmenopausal women, and Gremlin-1 showed inverse associations with IGF-1 and HDL cholesterol in unadjusted analyses. These relationships were most apparent in non-obese women, suggesting that Gremlin-1–metabolic associations may be more readily detectable in metabolically less complex states. However, when age was explicitly accounted for using age-adjusted partial correlations, the associations between Gremlin-1 and IGF-1 and between Gremlin-1 and HDL cholesterol were no longer statistically significant in either BMI stratum. This finding is consistent with the hypothesis that shared age-related variance largely explains the observed unadjusted associations, underscoring the dominant role of aging in shaping these endocrine–metabolic relationships.

The disappearance of Gremlin-1–IGF-1 and Gremlin-1–HDL associations after age adjustment has important mechanistic implications. IGF-1 is known to decline with advancing age and plays a central role in maintaining insulin sensitivity, lipid homeostasis, and tissue repair [24], and its hepatic production depends on intact GH and insulin signaling [25]. Similarly, HDL cholesterol is adversely affected by metabolic deterioration and may change with aging and menopausal transition. The present findings suggest that rising Gremlin-1 levels track alongside these age-related endocrine changes rather than exerting an independent effect on IGF-1 availability or lipid metabolism. Thus, Gremlin-1 may be better interpreted as a correlate of systemic aging processes, rather than a causal mediator linking adipose dysfunction or menopausal status to metabolic decline.

Pro-inflammatory cytokines such as IL-6 and TNF-α are known to suppress IGF-1 synthesis and signaling [24], while Gremlin-1 expression is increased in inflamed adipose and hepatic tissues [26,27]. In NAFLD/NASH, Gremlin-1 has been linked to cellular senescence and fibrotic remodeling [28]. whereas IGF-1 appears to exert antifibrotic and cytoprotective effects [29]. A reduction in IGF-1 may therefore weaken compensatory mechanisms that normally counterbalance Gremlin-1–associated tissue remodeling.

IGF-1 declines with advancing age and estrogen deficiency [30], whereas Gremlin-1 expression tends to increase in metabolically unfavorable conditions [8]. Although estrogen levels were not directly correlated with Gremlin-1 in our study, the inverse associations between age and IGF-1, and the correlation of age and Gremlin-1, suggest that postmenopausal endocrine changes may indirectly amplify Gremlin-1 signaling through loss of IGF-1-mediated metabolic protection. Elevated Gremlin-1 may therefore mark a more catabolic, insulin-resistant endocrine milieu in older women.

Finally, Gremlin-1 concentrations increased with age independently of BMI in multivariable models, supporting the interpretation that Gremlin-1 reflects qualitative aspects of metabolic aging, rather than adiposity alone. This observation is consistent with prior reports showing age-related increases in Gremlin-1 alongside impaired insulin signaling (7), as well as evidence that age may be a stronger determinant of adipose GREM1 expression than BMI (5). These findings support a potential role for Gremlin-1 in aging-related metabolic decline and highlight this pathway as a possible target for improving metabolic health in older women.

This study has several limitations. The relatively modest sample size may limit statistical power and generalizability of the findings. In addition, women in the perimenopausal transition were not included, restricting assessment of dynamic changes in Gremlin-1 across the full spectrum of reproductive aging. Although menopausal status was clinically defined, follicle-stimulating hormone (FSH) levels were not measured, which could have strengthened hormonal characterization. The cross-sectional design precludes causal inference, and the observed associations cannot establish temporal relationships.

Despite these limitations, an important contribution of the present study is the demonstration that circulating Gremlin-1 does not provide independent information beyond chronological age and established endocrine–metabolic markers, thereby refining its interpretation and limiting overextension of its biological significance.

## 5. Conclusions

The present findings are consistent with circulating Gremlin-1 primarily reflecting chronological aging and its associated endocrine–metabolic changes, rather than menopausal status or adiposity alone. While Gremlin-1 may participate in multiple tissue-specific aging processes, its systemic elevation appears to track with age-related declines in IGF-1 and metabolic resilience. Future longitudinal studies incorporating ovarian reserve markers, detailed adipose phenotyping, and repeated biomarker measurements will be necessary to clarify the tissue-specific origins, clinical significance, and potential biomarker role of circulating Gremlin-1 in women across the lifespan.

## Figures and Tables

**Table 1:** Descriptive analysis of the participants’ characteristics.

Variable		Mean (SD)
Age (years),		45.51 (13.47)
Body Height (cm)		157.20 (5.86)
Body weight (kg)		73.60 (16.75)
Body Mass Index (BMI) (kg/m^2^)		29.88 (6.99)
**Menopause Status**	**Frequency**	**Percentage**
Reproductive-aged	48	54.5
Postmenopausal	40	45.5

**Table 2:** Bivariate analysis of women’s menopausal status profiles.

	Menopause Status			
Laboratory Test	Reproductive-Aged, *n* = 48	Post-Menopausal, *n* = 40	t/df	*p*-Value
Age (years), mean (SD)	35.1 (0.96)	58 (5.92)	15.1/86	< 0.001
BMI, mean (SD)	28.42 (7.34)	31.64 (6.20)	2.20/86	0.031
Gremlin-1 [pg/mL]	124.05 (87.45)	251.84 (212.41)	3.56/86	0.001
Estradiol2 [pg/mL]	484.11 (570.65)	33.26 (13.50)	5.47/86	< 0.001
GH [ng/mL]	1.92 (2.77)	0.96 (1.67)	1.99/84	0.050
IGF-1 [ng/mL]	140.031 (55.40)	78.953 (27.79)	6.20/84	< 0.001
FBG [ mmol/L]	5.08 (1.82)	5.83 (1.03)	2.46/86	0.016
HbA1C [%)]	5.66 (0.81)	6.35 (0.93)	3.68/86	< 0.001
Total Insulin [µU/mL]	15.17 (15.12)	14.25 (11.63)	0.32/86	0.750
Cholesterol [mmol/L]	4.96 (1.01)	5.54 (4.81)	0.75/86	0.457
HDL [mmol/L]	1.53 (0.37)	1.39 (0.31)	1.96/86	0.053
LDL [mmol/L]	2.93 (0.91)	2.73 (0.88)	1.08/86	0.282
TG [mmol/L]	1.06 (0.52)	1.69 (1.76)	2.17/86	0.036

Abbreviations: BMI, body mass index; FBG, fasting blood glucose; HDL, high-density lipoprotein; LDL; low-density lipoprotein; TG, triglycerides; IGF-1, insulin-like growth factor-1; GH, growth hormone.

**Table 3:** Multivariable Generalized Linear analysis of women’s mean Gremlin-1 level score.

		95% CI for Beta		
	Beta Coefficient	Lower	Upper	*p*-Value
Intercept	−103.942	−280.338	72.454	0.245
Age (years)	5.968	1.302	10.634	0.013
menopause status = post-menopause	9.860	−113.725	133.444	0.874

Dependent outcome variable: mean Gremlin level. Probability distribution: Normal Link function: Identity.

**Table 4:** BMI-stratified unadjusted correlations between circulating Gremlin-1 and metabolic biomarkers.

BMI Category	*n*	Gremlin-1 vs. IGF-1 (r)	*p*-Value	Gremlin-1 vs. HDL (r)	*p*-Value
<30 kg/m^2^	41–42	−0.447	**0.003**	−0.340	**0.028**
≥30 kg/m^2^	45–46	−0.196	0.197	−0.023	0.881

Pearson correlation coefficients calculated within each BMI stratum using complete cases. Bold values indicate *p* < 0.05.

**Table 5:** Age-adjusted partial correlations between circulating Gremlin-1 and metabolic biomarkers, stratified by BMI category.

BMI Category	*n*	Gremlin-1 vs. IGF-1 (Partial r)	*p*-Value	Gremlin-1 vs. HDL (Partial r)	*p*-Value
<30 kg/m^2^	41	−0.094	0.557	−0.167	0.298
≥30 kg/m^2^	45	0.054	0.725	−0.006	0.968

Partial Pearson correlation coefficients adjusted for age and calculated within each BMI stratum using complete cases.

## Data Availability

The raw data supporting the conclusions of this article will be made available by the authors on request.

## Abbreviations

The following abbreviations are used in this manuscript:

GH	Growth Hormone
IGF-1	Insulin-like Growth Factor 1
HbA1c	Glycated Hemoglobin 1 c
E2	Estradiol2
